# Detection and characterization of novel respiratory viruses among native ducks (*Anas luzonica*) in Central Luzon, the Philippines

**DOI:** 10.5365/wpsar.2025.16.2.1124

**Published:** 2025-04-07

**Authors:** Milagros R Mananggit, Joely T Ongtangco, Xandre D Baccay, Ronnie Domingo, Mary Elizabeth Miranda, Romeo Gundran, Dan Drexel dela Cruz, Frank YK Wong, S Gabrielle Cody, Laura A Pulscher, Emily R Robie, Emily S Bailey, Gregory C Gray

**Affiliations:** a Department of Agriculture, Regional Field Office III, Pampanga, Philippines.; b Bureau of Animal Industry, Quezon City, Philippines.; c Field Epidemiology Training Program Alumni Foundation, Manila, Philippines.; d Central Luzon State University, Nueva Ecija, Philippines.; e CSIRO Australian Centre for Disease Preparedness, World Organisation for Animal Health Reference Laboratory for Avian Influenza, Geelong, Victoria, Australia.; f Division of Infectious Diseases, Department of Internal Medicine, University of Texas Medical Branch, Galveston, Texas, United States of America.; g Duke Global Health Institute, Duke University, Durham, North Carolina, United States of America.; h College of Pharmacy & Health Sciences, Department of Public Health, Campbell University, Lillington, North Carolina, United States of America.; i Institute for Human Infections and Immunity, and Departments of Internal Medicine (Infectious Diseases), Microbiology and Immunology, and Global Health, University of Texas Medical Branch, Galveston, Texas, United States of America.

## Abstract

**Objective:**

This cross-sectional, prospective surveillance study sought to determine the prevalence of novel respiratory viruses among domestic ducks in Central Luzon that are known to have frequent contact with wild avian species. Such contact may lead to novel virus spillover events that may harm domestic poultry as well as humans.

**Methods:**

From March 2019 to January 2020, cross-sectional and prospective surveillance for viruses among domestic ducks (*Anas luzonica*) was conducted by periodically collecting oropharyngeal swabs from ducks on 54 farms across three municipalities within Central Luzon (Region III). A flock of 30 sentinel domestic ducks was also sampled four times after being confined in the Candaba swamp. The resultant 1740 swab samples were pooled (5 samples/pool, 348 pools) by site and screened with molecular assays for respiratory viruses from multiple viral families.

**Results:**

Two farms yielded samples positive for avian influenza virus in Candaba, where adolescent ducks are known to freely mix with wild birds as they graze in rice fields. Overall, the prevalence of avian influenza virus was 2.3% (8/348 pools). Sequencing revealed three pools with highly pathogenic avian influenza H5N6, one with low pathogenicity H5N8, and one with H5 with an unspecified neuraminidase. All the pooled specimens tested were negative for influenza C, adenoviruses, coronaviruses and enteroviruses.

**Discussion:**

Although this study had several limitations, it found supportive evidence that domestic ducks are acquiring avian influenza viruses from wild bird species. These findings underscore recommendations that duck farmers should seek to prevent domestic ducks from mixing with wild avian species.

Respiratory viruses are common among avian species. Wild birds are thought to serve as reservoirs that move novel respiratory viruses across geographical areas and introduce such viruses to livestock species. (*1*-*3*) Transmission is accelerated by wild bird migrations, movements of commercial poultry, and close contact between various live bird species in wet markets. (*1*, *2*)

Coronaviruses, adenoviruses and enteroviruses are known to cause disease among domestic and wild bird species, often resulting in severe morbidity. (*4*-*6*) Of particular public health importance are avian influenza viruses (AIVs), specifically some H5 and H7 subtypes, which occasionally cause illness among humans and other animal species. (*7*) The highly pathogenic influenza H5N6 virus was first identified in 2013, and infection with this virus includes severe clinical symptoms and mortality across avian species. (*8*) Forty-seven countries reported AIVs among humans or avian species between December 2022 and June 2023. (*9*)

In the Philippines, highly pathogenic AIV was first reported in July 2017, when the identification of H5N6 in chickens and quails at egg farms in Central Luzon resulted in the culling of more than 400 000 poultry within 1 km of the outbreak sites. (*10*, *11*) At the time, agriculture officials suspected that the source of the outbreak stemmed either from the interaction of domestic birds with migratory waterfowl in the Candaba swamp or the smuggling of live ducks from China. (*12*) To date, neither hypothesis has been proven. (*11*) This study sought to identify respiratory viruses of interest among domestic duck populations across Central Luzon that may point towards viral transmission between wild avian species and domestic ducks.

## Methods

### Collaboration, recruitment and sample collection

**Fig. 1:**
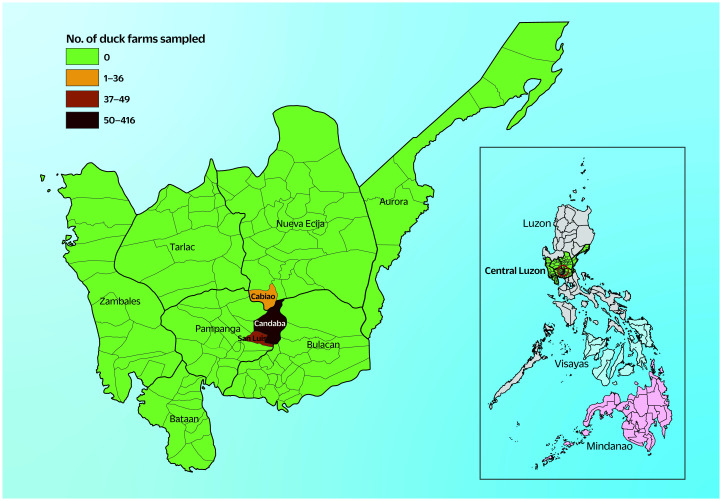
Map of Central Luzon/Region III, Philippines, showing the municipalities (Cabiao, Nueva Ecija province; Candaba and San Luis, Pampanga province) where the sampled duck farms were located

Duck farms were selected from Cabiao municipality in Nueva Ecija province and Candaba and San Luis municipalities in Pampanga province, Central Luzon (**Fig. 1**), by drawing lots from the list of facilities raising ducks in each barangay (i.e. district or ward) and municipality (**Table 1**). Selected farms were incentivized to participate in the study through the provision of  water-soluble vitamin supplements for their ducks and offers to provide the results of laboratory tests for free. No ducks were vaccinated against the respiratory viruses investigated in this study.

[**insert** Figure 1]

**Table 1:** Number of duck farms selected, specimens collected and results of testing for influenza A virus, by municipality, Central Luzon, Philippines, March 2019 and January 2020

Municipality, province	No. of duck farms in municipality	No. of farms selected	No. of samples collected	No. of pooled samples	No. positive for influenza A virus
**Candaba, Pampanga**	**416**	**45**	**1350**	**270**	**8**
**Candaba swamp ** **(sentinel ducks)**	**N/A**	**1**	**120a**	**24**	**0**
**San Luis, Pampanga**	**49**	**5**	**150**	**30**	**0**
**Cabiao, Nueva Ecija**	**36**	**4**	**120**	**24**	**0**
**Total**	**501**	**55**	**1740**	**348**	**8**

N/A: not applicable.

^a^ Samples were collected from each of 30 sentinel ducks after their introduction to the Candaba swamp on days 1, 10, 20 and 30.

In each of the 54 selected duck farms, the field team explained the study to farm owners and workers, then collected oropharyngeal swabs from 30 birds on each farm using flexible sterile applicator swabs. Workers were asked to catch and sample representative ducks in every pen, for a total of 30 ducks per farm. Samples were placed in a viral transport medium, labelled, stored in a cooler with ice and transported the same day to Regional Field Office III of the Regional Animal Disease Diagnostic Laboratory (RADDL) at the Department of Agriculture, Pampanga. The study team also collected descriptive data about the farm, the ducks on each farm and duck-grazing habitats.

To determine whether the source of the first bird flu outbreak in the Philippines in 2017 was from migratory birds, a flock of 30 sentinel ducks was purchased and placed in the Candaba swamp. They were fenced in with netting and sampled four times, 10 days apart, during the height of the migration season (November to December 2019), using the same methods as described above.

Researchers and farm workers wore appropriate personal protective equipment (PPE) including scrub suits, laboratory gowns, face masks, face shields, gloves and boots during sampling. Disposable PPE and used applicator swabs were autoclaved in the laboratory before disposal. Nondisposable PPE was cleaned and disinfected.

### Laboratory testing

Nucleic acid extraction was performed using the QIAamp MinElute Virus Spin Kit (Qiagen, Germantown, MD, USA) at the Regional Avian Influenza Diagnostic Laboratory (located at RADDL), according to the manufacturer’s instructions. Five field samples from the same farm were combined into one pool and vortexed. RNA from each pool was extracted and aliquoted into three tubes for matrix gene detection, haemagglutinin subtyping and characterization, and then stored at −80 °C.

The pooled samples were screened for influenza A and C viruses, adenoviruses (pan-species), coronaviruses (pan-species) and enteroviruses (pan-species) at RADDL in Central Luzon using quantitative reverse transcription–polymerase chain reaction (qRT–PCR) protocols provided by Duke University. (*13*) Samples positive for influenza A were sent to the Research Institute for Tropical Medicine and the University of the Philippines to determine the haemagglutinin subtype.

RNA extracted from the pooled samples, which tested positive for influenza A virus, was shipped to the CSIRO Australian Centre for Disease Preparedness (ACDP) for confirmation and further characterization. When the results of the molecular assays were discordant between the Philippine and Australian laboratories, the results from the ACDP were reported, as it is a World Organization for Animal Health reference laboratory for avian influenza.

## Results

### Samples

A total of 1740 oropharyngeal swabs were collected between March 2019 and January 2020, resulting in 348 pooled samples from 54 domestic duck farms and the sentinel ducks inserted in the Candaba swamp. Of these, 24 pooled samples were from the sentinel ducks (**Table 1**). All ducks appeared healthy when samples were collected.

### Influenza A virus

Of the 348 pooled samples, 8 (2.3%) were positive for influenza A virus (**Table 1**). These positive pooled samples were from two farms in two different barangays in Candaba, Pampanga. Infected ducks in these pooled samples were aged 6–8 months, and all had a history of grazing in the rice field (**Tables 2**,**3**).

**Table 2:** Results of molecular assay for influenza A virus, by age of duck, Central Luzon, Philippines, March 2019 and January 2020

Age of duck	No. of farms	No. of farms with positive samples	No. of farms with negative samples
**5 months**	**8**	**0**	**8**
**6 months**	**34**	**1**	**33**
**8 months**	**13**	**1**	**12**
**Total**	**55**	**2**	**53**

**Table 3:** Haemagglutinin typing results for samples with influenza A viruses (*n* = 8), by farm with corresponding grazing area, Central Luzon, Philippines, March 2019 to January 2020

Laboratory ID of the farm (sample pool no.)	Haemagglutinin subtype	Grazing area	Age of duck (months)
**1754 (4)**	**H5**	**Rice field**	**8**
**1754 (2)**	**H5**	**Rice field**	**8**
**289 (1)**	**H5**	**Rice field**	**6**
**289 (2)**	**H5**	**Rice field**	**6**
**289 (3)**	**Not determined**	**Rice field**	**6**
**289 (4)**	**H5**	**Rice field**	**6**
**289 (5)**	**H5**	**Rice field**	**6**
**289 (6)**	**H5**	**Rice field**	**6**

The 24 pooled samples from the sentinel ducks were all negative for influenza A virus (**Table 1**). Thus, they did not acquire AIV during the 30 days of sampling in the Candaba swamp.

In haemagglutinin characterization, 7 of the 348 (2.0%) pooled samples were positive for avian influenza H5 (**Table 3**). Next-generation sequencing confirmed the presence of clade 2.3.4.4 highly pathogenic H5N6 in several samples (GISAID EpiFlu accession numbers EPI3467823–EPI3467846), with one additional sample having evidence of low pathogenicity avian influenza H5N8 (**Table 4**), GISAID EpiFlu accession numbers EPI3467819–EPI3467822. The H5 subtype detected was similar to other related viruses previously identified in the Philippines.

**Table 4:** Sequence typing results for influenza A viruses detected at duck farms, Central Luzon, Philippines, March 2019 and January 2020

Laboratory ID of the farm	Sample pool no.	Test		Sequence results
		Avian influenza virus type A	Avian influenza virus H5	
**1754**	**2**	**Positive**	**Positive**	**Undetected**
**1754**	**4**	**Positive**	**Positive**	**Positive for H5N8**
**289**	**1**	**Positive**	**Positive**	**Undetected**
**289**	**2**	**Positive**	**Positive**	**Positive for H5N6**
**289**	**3**	**Positive**	**Negative**	**Undetected**
**289**	**4**	**Positive**	**Positive**	**Positive for H5**
**289**	**5**	**Positive**	**Positive**	**Positive for H5N6**
**289**	**6**	**Positive**	**Positive**	**Positive for H5N6**

### Other respiratory pathogens

Only 316 pooled samples were tested for adenovirus with the qRT–PCR assay due to insufficient reagents. None of the pooled samples yielded molecular evidence of influenza C virus, coronaviruses, adenoviruses or enteroviruses.

## Discussion

Of the 54 participating farms and the sentinel duck site in Central Luzon, samples from two farms were positive for influenza A virus. Eight (2.3%) of the 348 pools were positive for influenza A. These positive specimens were obtained from ducks aged 6–8 months with a history of grazing in rice fields after harvest season, allowing them to mix with wild birds that were also feeding in the rice fields. Duck farmers often pasture young ducks in rice fields and other bodies of water where migratory birds may reside to lessen the cost of feed and to control golden snails, other pests and insects infesting these bodies of water. When ducks begin to lay eggs, they are confined to laying houses that are often open to wild bird incursions (personal communication with the Provincial Veterinary Office of Pampanga).

Despite detecting AIV in farmed ducks, the molecular studies for the 30 sentinel ducks were all negative for influenza A virus, thus transmission of AIV from wild birds to sentinel ducks in the Candaba swamp could not be demonstrated during their 30-day stay in the area. This could be due to the limited time and space that the sentinel birds had to mix with wild birds due to being confined in netting.

The sequencing results from ACDP identified multiple strains of highly pathogenic avian influenza among the surveyed flocks, including an H5N6 strain similar to a strain associated with the 2017 outbreak. This is of particular concern, as low levels of circulating highly pathogenic AIV may quickly lead to additional large-scale outbreaks, with catastrophic consequences, if not properly identified and curtailed. The 2017 outbreak caused public panic due to fear of humans becoming infected by consuming duck eggs or meat. The additional H5N6 outbreaks in 2020, along with the identification of H5N1 in Pampanga in 2022 (*14*) and again in 2023, (*15*) attest to the difficulty of eliminating AIVs once they have become enzootic in domestic livestock. While all ducks appeared healthy during sampling, the presence of AIVs in these populations presents the possibility that these ducks could asymptomatically transmit avian influenza A viruses to chickens or quail housed at nearby farms.

The ducks in this study were not vaccinated against AIV or the other respiratory viruses investigated. In late 2023, the Philippine Department of Agriculture issued guidelines on targeted AIV vaccination to pre-empt future outbreaks and complement the existing vaccination programme. (*16*) However, AIV vaccines are not part of the mandated vaccination schedule for domestic poultry and remain at the discretion of the farmer.

This study had several limitations. We sampled ducks from only 55 sites in Central Luzon and could have missed important circulating AIVs. We used only oropharyngeal sampling to prevent further stress on laying ducks and for ease of collection. Our sentinel duck experiment could have failed due to the short period (30 days) the ducks were exposed to wild birds and also due to their confinement within netting that limited their mixing with wild birds.

Despite these limitations, the findings are consistent with the notion that wild birds are introducing AIVs to farmed ducks. These findings underscore recommendations from the Government of the Philippines that duck farmers should protect domestic ducks from contact with wild birds.
